# Real‐world prevalence of the inclusion criteria for the LEADER trial: Data from a national general practice network

**DOI:** 10.1111/dom.13710

**Published:** 2019-04-11

**Authors:** William Hinton, Michael Feher, Neil Munro, Megan Walker, Simon de Lusignan

**Affiliations:** ^1^ Department of Clinical and Experimental Medicine University of Surrey Guildford UK; ^2^ Medical Affairs Department, Novo Nordisk Ltd Gatwick UK; ^3^ Royal College of General Practitioners Research and Surveillance Centre London UK

**Keywords:** cardiovascular disease, incretin therapy, liraglutide, primary care, type 2 diabetes

## Abstract

**Aims:**

To explore the prevalence and describe the clinical characteristics of people with type 2 diabetes with a similar cardiovascular (CV) profile to that of the LEADER trial participants in a primary care setting in England.

**Materials and methods:**

In this cross‐sectional analysis, using the Royal College of General Practitioners (RCGP) Research and Surveillance Centre (RSC) network database, we identified people with type 2 diabetes meeting the LEADER inclusion criteria. We identified people's CV risk factors using computerized medical records. Additionally, we assessed the prescription pattern of glucagon‐like peptide‐1 receptor agonists (GLP‐1RAs) in this cohort.

**Results:**

Of 1 275 461 adults, we identified 84 394 with type 2 diabetes, of whom 14 000 (16.6%) met the LEADER inclusion criteria for established or high‐risk CV disease (RCGP RSC‐CVD group). The LEADER cohort was younger than the RCGP RSC‐CVD group (64.2 vs 73.2 years), had higher mean glycated haemoglobin (71.6 vs 67.1 mmol/mol) and blood pressure (BP) values (systolic BP: 135.9 vs 132.9 mmHg; diastolic BP: 77.2 vs 72.7 mmHg), and a higher mean body mass index (32.5 vs 30.9 kg/m^2^). In the RCGP RSC‐CVD group, only 1215 people (8.7%) had ever been prescribed a GLP‐1RA and 760 (5.4%) had ever received liraglutide.

**Conclusions:**

In a cohort of English general practice patients, one in six people with type 2 diabetes met the LEADER inclusion criteria, and less than one in 10 of these received liraglutide, a drug which has demonstrated CV benefits amongst others. There is scope to improve the outlook in people with type 2 diabetes and high CV risk through evidence‐based use of specific GLP‐1RAs.

## 1 INTRODUCTION

Cardiovascular outcomes trials (CVOTs) were established after the 2007 report on rosiglitazone, in which concerns were raised over the cardiovascular (CV) safety of type 2 diabetes therapies.^1^ In 2008, the US Food and Drug Administration issued guidance to the pharmaceutical industry, requiring additional data to demonstrate CV safety for new glucose‐lowering therapies in patients with type 2 diabetes.^2^ The trials were designed to assess the safety of antidiabetic treatments compared to placebo with respect to major adverse CV events (MACE), including CV death, stroke and non‐fatal myocardial infarction (MI).

To date, 17 randomized CVOTs have been completed, and all have confirmed non‐inferiority for the therapies assessed in terms of CV safety when compared with placebo.^3^, ^4^, ^5^, ^6^, ^7^, ^8^, ^9^, ^10^, ^11^, ^12^, ^13^, ^14^, ^15^, ^16^, ^17^, ^18^, ^19^ In addition, five of the 17 completed CVOTs have also confirmed significant reductions in the primary MACE composite endpoint for the sodium‐glucose co‐transporter‐2 (SGLT2) inhibitors empagliflozin and canagliflozin,^8^, ^14^ and for the glucagon‐like peptide‐1 receptor agonists (GLP‐1RAs) liraglutide, albiglutide and dulaglutide,^16^, ^18^, ^19^ suggesting that these agents have direct cardioprotective properties. A sixth study, the SUSTAIN 6 trial, reported CV superiority compared to placebo for the GLP‐1RA semaglutide in a post hoc analysis.^15^

In the LEADER trial, the CV effects of the GLP‐1RA liraglutide were assessed in patients at high CV risk, and the results confirmed a significant 13% reduction in MACE and 15% reduction in death from any cause for patients treated with once‐daily liraglutide compared with placebo, when both were added to standard care.^16^

In addition to randomized clinical trials, there is increasing interest in the use of real‐world evidence.^20^ Whilst CVOT results have undoubtedly provided valuable information about the CV safety separate from glucose‐lowering benefits, the inclusion criteria in a specific CVOT, and therefore its results, may apply to only a small percentage of patients with type 2 diabetes, and consequently may not be generalizable to the wider clinical population.

The aims of the present study were, firstly, to evaluate the extent to which the LEADER trial population can be identified in a real‐world cohort by assessing the prevalence of patients with type 2 diabetes in an English general practice setting who possess the same inclusion profile as those recruited to the LEADER trial and, secondly, to assess the pattern of GLP‐1RA prescription in this cohort.

## 2 MATERIALS AND METHODS

The study was a cross‐sectional analysis of all people with type 2 diabetes included in the Royal College of General Practitioners (RCGP) Research and Surveillance Centre (RSC) network database, conducted to quantify the proportion of people with CV disease or at high CV risk meeting the eligibility criteria for the LEADER trial.

The RCGP RSC database is a primary care sentinel network. It uploads computerized medical records twice weekly from over 200 primary care practices distributed across England, producing near real‐time reports about influenza, vaccine effectiveness and, more recently, other areas of research.^21^ Covering, at the time of the present study, a population of >2 000 000 patients, the RCGP RSC provides a representative sample of the national English population in terms of demographics and clinical outcomes.^22^, ^23^ As a registration‐based system, where patients are identified with a unique National Health Service (NHS) number, the RCGP RSC database enables capture of a representative population without double counting.

The full methods of the study protocol have been previously published.^24^ Using the RCGP RSC database, people with type 2 diabetes who had a similar CV risk profile to those included in the LEADER trial (RCGP RSC‐CVD group) were identified and compared with the liraglutide‐treated group from the LEADER trial. Within the RCGP RSC‐CVD group, the proportion of patients who had ever been prescribed at least one of the following GLP‐1RAs was determined: albiglutide, dulaglutide, exenatide, exenatide extended release, liraglutide and lixisenatide. Data for the once‐weekly GLP‐1RA semaglutide could not be collected, as the treatment was not commercially available in the United Kingdom at the time of the analysis.

### 2.1 Data analysis

In this analysis, data were extracted for all patients from the RCGP RSC database for information collected up to December 31, 2016, comprising all patients with type 2 diabetes who were aged ≥18 years on or before this date.

People with type 2 diabetes were identified using a two‐step ontology‐based process.^25^ Firstly, we identified individuals with diabetes using a combination of diagnostic codes, glycated haemoglobin (HbA1c) and blood glucose test results, and antihyperglycaemic therapy usage (except metformin). Subsequently, people with diabetes were categorized by diabetes type using a seven‐step algorithm that takes into account medication history, diagnosis codes and other key clinical characteristics.

The RCGP RSC‐CVD subgroup was identified from the cohort of people with type 2 diabetes by using clinical codes (Tables S1–S12). These coded data included diagnosis and treatment information, prescriptions and laboratory data.^26^ Individuals were considered to fulfill the LEADER criteria if they had any of the disease‐defining codes at any time in their clinical record. To compare the type 2 diabetes group with the LEADER inclusion criteria for CV disease or risk, we used the closest matching variables available from routine UK primary care data and, when specific conditions were not recorded in primary care data (due to coding limitations or non‐specific data entry), broader criteria were used (Table S13).

We reported the clinical characteristics of the RCGP RSC‐CVD group. These included age, gender, duration of diabetes, HbA1c, body mass index (BMI), systolic blood pressure (BP) and diastolic BP. Age was reported as per the end of the study period (December 31, 2016). Duration of diabetes was based on the first indicator of diabetes in the patient's record up to the end of the study period. HbA1c, BMI, systolic and diastolic BP were taken from the latest patient's record. Clinical characteristics of the cohorts are described using descriptive statistics (percentages, means and SD values).

### 2.2 Compliance with ethics guidelines

Approval for use of the data was acquired from the RCGP RSC Study Approval Committee. This study did not require ethical approval as it was considered to be a clinical audit when tested against the Health Research Authority/Medical Research Council tool “Is my study research?”.^27^ Part of our standard way of working is not to process the data of people who have opt‐out codes; these affect 2.25% of our population.^28^

## 3 RESULTS

At the time of data extraction, the RCGP RSC population comprised 1 275 461 adults. From this population, we identified 84 394 (6.6%) people with type 2 diabetes. Of those with type 2 diabetes, 14 000 (16.6%) met the LEADER trial inclusion criteria (Table 1) for either CV disease or CV risk (RCGP RSC‐CVD group).

**Table 1:** Inclusion criteria for the LEADER trial

**LEADER trial inclusion criteria** 16
Type 2 diabetes with HbA1c ≥ 7.0% is 53.0 mmol/mol
CV disease group: age ≥50 years and ≥1 of the following: Previous MI Previous stroke or transient ischaemic attack Previous coronary, carotid or peripheral arterial revascularization >50% stenosis of coronary, carotid or lower extremity arteries History of symptomatic CHD documented by positive exercise stress test or any cardiac imaging or unstable angina with ECG changes Asymptomatic cardiac ischaemia documented by positive nuclear imaging test, exercise test or dobutamine stress echocardiogram Chronic heart failure NYHA class II–III Chronic renal failure: eGFR <60 mL/min/1.73 m^2^ (MDRD formula) eGFR <60 mL/min (Cockcroft–Gault formula)
No previous CV disease group: age ≥60 years and ≥1 of the following:
Microalbuminuria (ACR) or proteinuria Hypertension and left ventricular hypertrophy by ECG or imaging Left ventricular systolic or diastolic dysfunction by imaging Ankle–brachial index <0.9

Abbreviations: ACR, albumin to creatinine ratio; CHD, coronary heart disease; CV, cardiovascular; eGFR, estimated glomerular filtration rate; HbA1c, glycated haemoglobin; MDRD, modification of diet in renal disease; MI, myocardial infarction; NYHA, New York Heart Association.

The liraglutide‐treated LEADER cohort was younger than the RCGP RSC‐CVD subgroup (64.2 vs 73.2 years, respectively), had a higher mean HbA1c (71.6 vs 67.1 mmol/mol), higher BP values (systolic BP: 135.9 vs 132.9 mmHg; diastolic BP: 77.2 vs 72.7 mmHg), and a higher mean BMI (32.5 vs 30.9 kg/m^2^; Table 2).

**Table 2:** Baseline characteristics of the RCGP RSC cardiovascular disease risk‐matched group compared with the liraglutide‐treated group from the LEADER trial

Characteristic	RCGP RSC‐CVD group (N = 14 000)	LEADER group (N = 4668)^16^
Age, years	73.2 (9.8)	64.2 (7.2)
Male, n (%)	8537 (61.0)	3011 (64.5)
Duration of diabetes, years	13.4 (8.0)	12.8 (8.0)
HbA1c, mmol/mol	67.1 (15.3)	71.6 (17.5)
BMI, kg/m^2^	30.9 (6.2)	32.5 (6.3)
SBP, mmHg	132.9 (15.4)	135.9 (17.8)
DBP, mmHg	72.7 (9.5)	77.2 (10.3)
**Established CV disease (age ≥ 50 years), n (%)**	**11 241 (80.3)**	**3831 (82.1)**
Prior MI	2717 (19.4)	1464 (31.4)
Prior cerebrovascular events	2623 (18.7)	730 (15.6)
Prior revascularization	2723 (19.5)	1835 (39.3)
>50% stenosis of coronary, carotid or lower extremity arteries	3882 (27.7)	1188 (25.4)
Documented symptomatic CHD	460 (3.3)	412 (8.8)
Documented asymptomatic cardiac ischaemia	NA	1241 (26.6)
Heart failure	1886 (13.5)	653 (14.0)
Chronic kidney disease^a^	6000 (42.9)	1185 (25.4)
**CV disease risk factors (age ≥60 years), n (%)**	**2759 (19.7)**	**837 (17.9)**
Microalbuminuria or proteinuria	2628 (18.8)	501 (10.7)
Hypertension and left ventricular hypertrophy	140 (1.0)	248 (5.3)
Left ventricular systolic or diastolic dysfunction	43 (0.3)	203 (4.3)
Ankle‐brachial index <0.9	9 (0.1)	110 (2.4)

Data are mean (SD) unless otherwise stated.

Abbreviations: BMI, body mass index; CHD, coronary heart disease; CV, cardiovascular; eGFR, estimated glomerular filtration rate; DBP, diastolic blood pressure; HbA1c, glycated haemoglobin; MI, myocardial infarction; n, number of patients with an event; NA, not available; RCGP RSC‐CVD group, Royal College of General Practitioners Research and Surveillance Centre population with established or high‐risk CV disease; SBP, systolic blood pressure.

a eGFR <60 mL/min/1.73 m^2^ per Modification of Diet in Renal Disease formula.

Approximately 80% of people in the RCGP RSC‐CVD group had established CV disease and 20% were at high CV risk; there was a similar split between established CV disease and those at high CV risk within the liraglutide‐treated LEADER cohort (Table 2). A larger proportion of patients from the liraglutide‐treated LEADER cohort had prior MI and coronary heart disease compared with the RCGP RSC‐CVD group; however, the same subgroup from the RCGP RSC cohort included a larger proportion of patients with prior cerebrovascular events and chronic kidney disease (estimated glomerular filtration rate [eGFR] <60 mL/min/1.73 m^2^; Table 2).

Within the RCGP RSC‐CVD group, 1215 patients (8.7%) had a prescription for a GLP‐1RA at some point in their medical records, and 760 (5.4%) had previously been treated with liraglutide.

## 4 DISCUSSION

The present study ascertained the prevalence of people with type 2 diabetes possessing a similar CV profile to those included in the LEADER CVOT, and also determined the pattern of prescribing of GLP‐1RAs in this cohort. The analysis showed that one in six people (16.6%) with type 2 diabetes in an English primary setting meets the inclusion criteria for the LEADER trial, and that <10% of the RCGP RSC‐CVD group had ever been prescribed a GLP‐1RA.

In the LEADER trial, the cardioprotective properties of liraglutide were a key clinical finding; however, the applicability of these results to the wider real‐world population is uncertain. Our study investigated whether the LEADER population could be identified in the real world; however, the eligibility criteria from the LEADER trial may not necessarily correspond with what is considered to be a clinically relevant high CV risk. In fact, although younger and with better renal parameters, patients from the LEADER trial were more likely to have had their CV disease detected and had subsequent surgical intervention than the real‐world RCGP RSC‐CVD cohort. Although patients in LEADER were primarily from a secondary care setting, within the United Kingdom there is a universal general practitioner registration system and all diabetes‐related data, wherever obtained, are collated in primary care, and therefore the differences in patient characteristics between the groups are unlikely to be a result of the primary care setting, but may reflect the multinational status of LEADER, as only 453 patients out of 9340 were from the United Kingdom.^29^ While it is possible that >16.6% of the wider English type 2 diabetes population are at high CV risk, the landmark UK Prospective Diabetes Study reported a figure of 20%, that is, a similarly low proportion of patients with macrovascular complications and comparable diabetes duration, suggesting that the LEADER inclusion criteria captured the majority of these patients.

From the completed CVOTs, at the time of the present analysis, liraglutide was the most widely commercially available GLP‐1RA in the United Kingdom that had shown CV superiority to placebo.^16^, ^30^ Other GLP‐1RA CVOT data have since been published^15^, ^18^, ^19^; however, given the label and commercial availability of other GLP‐1RAs, we have kept the focus of this publication on liraglutide. In 2016, when the results from LEADER were published, prescription of new medications, such as liraglutide, was guided and healthcare providers in the United Kingdom were restricted by national and regional recommendations when deciding on a treatment option for their patients. This, in addition to other factors, such as cost, lack of knowledge, acceptance of an injectable medication, and clinical inertia, may have resulted in a low prescription rate for GLP‐1RAs, including liraglutide, in those people with type 2 diabetes and high CV risk. Presently, in light of the positive CVOT results seen with GLP‐1RAs, a more individualized approach to diabetes management may prevail.

Currently, the National Institute for Health and Care Excellence (NICE) treatment algorithm for type 2 diabetes recommends use of GLP‐1RAs as a fourth‐line therapy, as add‐on to metformin and sulphonylureas, in people with a BMI ≥35 kg/m^2^ (adjusted accordingly for people from black, Asian and other minority ethnic groups) or a BMI <35 kg/m^2^, and for whom insulin therapy would have significant occupational implications, or weight loss would benefit other significant obesity‐related comorbidities.^31^ NICE recommendations for type 2 diabetes, however, were published in 2015, and therefore do not reflect the recent evidence from CVOTs. Furthermore, these guidelines only recommend drug classes rather than individual agents, which may not take into account the recent developments within the GLP‐1RA class in terms of CV benefits.^32^ More recently, other national and international guidelines have started recognizing the latest CVOT results and, as a consequence, many of these guidelines now recommend use of medications with proven CV benefit earlier in the treatment pathway in people with type 2 diabetes who are at high risk of CV disease.^33^, ^34^ For example, in 2017, the Scottish Intercollegiate Guidelines Network (SIGN) recommended the use of SGLT2 inhibitors and GLP‐1RAs with proven CV benefit in people with type 2 diabetes and established CV disease.^34^ In addition, the American Diabetes Association (ADA) and the European Association for the Study of Diabetes (EASD) released a joint consensus, whereby the first decision point in establishing the most appropriate treatment for patients with type 2 diabetes is to evaluate whether patients have established atherosclerotic CV disease (ASCVD) or chronic kidney disease. In those patients with type 2 diabetes and established ASCVD, or in those with established chronic kidney disease or heart failure, the consensus recommends use of GLP‐1RAs or SGLT2 inhibitors with proven CV benefits.^35^

Based on the number‐needed‐to‐treat from the LEADER trial,^16^ if all the patients in the RCGP RSC‐CVD group were treated with liraglutide, the number of MACE that could be prevented over 3 years would be 212. This calculation may be an overestimate as it does not take into account the numbers of individuals from the RCGP RSC‐CVD group who may be already treated with other antidiabetic therapies, such as the SGLT2 inhibitors empagliflozin and canagliflozin, which have also reported CV benefits; however, it suggests that there is scope for offering more personalized therapies with proven CV benefit to achieve better health outcomes. There is increasing clinical evidence to suggest that GLP‐1RAs may offer further benefits beyond that of cardioprotection, such as increasing satiety and reducing overweight and obesity,^36^, ^37^, ^38^, ^39^ which may confer relevant lifetime CV risk reductions in overweight or obese people with type 2 diabetes without CVD. More evidence on the longer‐term use of GLP‐1RAs is needed.

Similar findings to those of the present study were shown in two studies describing the real‐world use of glucose‐lowering therapies in patients at high CV risk using Scottish and United States‐based databases. Using a national registry linked to hospital admissions, a Scotland‐based study reported a higher percentage of patients with type 2 diabetes and established CV disease (44% of the total type 2 diabetes population, based on the LEADER inclusion criteria), and yet only 2.4% of this cohort were treated with a GLP‐1RA.^40^ Similarly, data from the US Diabetes Collaborative Registry showed that 48% of people with type 2 diabetes from a primary or secondary care setting would meet the same eligibility criteria for LEADER, but only 6% of these were prescribed a GLP‐1RA.^41^ While it could be argued that the slow uptake of GLP‐1RAs may be attributable to the reluctance of physicians to initiate injectable treatments in their patients with type 2 diabetes, analogous studies with oral glucose‐lowering treatments with proven CV benefits, such as the SGLT2 inhibitor empagliflozin, showed a similar pattern to that observed with GLP‐1RAs. A study assessing the EMPA‐REG CVOT inclusion criteria against the RCGP RSC cohort database reported that the inclusion criteria are applicable only to a small proportion of people with type 2 diabetes (15.7% of the total type 2 diabetes population), and that an even smaller proportion of those who are currently treated with SGLT2 inhibitors have the same high CV risk as that of the EMPA‐REG trial population (11.1% of the total type 2 diabetes population), thus calling into question whether this class of drug is also being used to its potential, at least in England.^42^

In addition, as the pre‐approval CVOT SUSTAIN 6 shares identical inclusion criteria to LEADER,^15^ the same RCGP RSC‐CVD cohort would be identified as in the present study, and may also benefit from the use of the once‐weekly GLP‐1RA semaglutide, when this is launched in the United Kingdom.

Key strengths and limitations of the data source used in the present study have been reported previously.^22^, ^25^ Several factors may limit the identification of the LEADER inclusion criteria risk factors from routine primary care data. The number of people meeting the LEADER eligibility criteria may be underestimated as a consequence of limited CV detection/intervention in clinical practice. Identification of specific inclusion criteria is limited by the clinical coding system (Read codes) used in UK primary care, which does not have a code that mapped directly onto each LEADER criterion, thus potentially leading to overestimation/underestimation of people meeting the inclusion criteria. Furthermore, missing cases may result from loss of information recorded as free text in the primary care database. It is also important to note that the LEADER trial mostly enrolled patients from the secondary care setting across the globe and, as such, the present analysis may underestimate the real representativeness of LEADER as a whole. Additionally, in the present analysis, rates of prior MI and prior revascularization were substantially lower in the RSCGP RSC‐CVD cohort than those in the LEADER cohort, whereas rates of prior cerebrovascular events and chronic kidney disease were higher. These results reflect a real difference between the two populations, as the same measurements were used to define each disease state in both patient groups. A major contributing factor to these differences may be the older age and slightly longer duration of diabetes in the RCGP RSC population. Generally, older people tend to have more comorbidities than younger ones; however, the latter are usually more represented in clinical trials. It should also be noted that the present findings regarding the differences in the populations between the English national general practice network database and LEADER do not reflect or predict in any way the likely efficacy and safety of liraglutide that could be expected in the wider population. Finally, full CVOT results and label updates reporting CV benefits associated with relevant therapies have only been recently published; therefore, it might still be too early to notice a change in the prescription pattern for GLP‐1RAs.

There would be scope through near real‐time networks such as the RCGP RSC to conduct studies monitoring the use of medications and to report CV outcomes, with practice fed back through our dashboard technology,^43^ to analyse how many patients might benefit from a change in treatment.

In conclusion, the LEADER study was the first CVOT to show CV benefits of GLP‐1RA therapy. Although only a small proportion of patients with type 2 diabetes from the English national general practice network database fulfil the stringent LEADER inclusion criteria, the majority of these patients have not been prescribed a GLP‐1RA.

With the wider acceptance of the results of CVOTs to guide more personalized or tailored therapy for people with type 2 diabetes, as highlighted in the recent ADA/EASD consensus,^33^ there is scope to improve the management of people at high CV risk through targeted use of therapies with a proven CV benefit.

## CONFLICT OF INTEREST

M.F. receives financial support for research, speaker meetings and consultancy from AMGEN, MSD, Merck, AstraZeneca, Pfizer, Novo Nordisk Ltd, Eli Lilly and Co. and Sanofi‐Aventis. W.H. receives research funding from Eli Lilly and Co., Novo Nordisk Ltd and AstraZeneca UK Ltd. N.M. has received financial support for research, speaker meetings and consultancy from MSD, Merck, BMS, AstraZeneca, Pfizer, Novo Nordisk Ltd, Eli Lilly and Co. and Sanofi‐Aventis. M.W. is an employee of Novo Nordisk Ltd. S.d.L. receives research funding via the University of Surrey from Eli Lilly Co., GlaxoSmithKline, Takeda, AstraZeneca and Novo Nordisk Ltd.

## AUTHOR CONTRIBUTIONS

W.H., M.F., N.M. and S.d.L. made substantial contributions to conception and design, and collected and analysed the data; M.W. substantially contributed to data interpretation. All authors were involved in drafting the manuscript and revising it for intellectual content, and have given final approval of the version to be published.

## Supporting information

Table S1. 5‐byte version 2 Read codes used to identify the presence of myocardial infarction.
Table S2. 5‐byte version 2 Read codes used to identify the presence of stroke and transient ischaemic attack.
Table S3. 5‐byte version 2 Read codes used to identify the presence of coronary artery disease.
Table S4. 5‐byte version 2 Read codes used to identify the presence of unstable angina.
Table S5. 5‐byte version 2 Read codes used to identify the presence of chronic heart failure.
Table S6. 5‐byte version 2 Read codes used to identify the presence of creatinine.
Table S7. 5‐byte version 2 Read codes used to identify the presence of albumin to creatinine ratio.
Table S8. 5‐byte version 2 Read codes used to identify the presence of hypertension.
Table S9. 5‐byte version 2 Read codes used to identify the presence of left ventricular hypertrophy.
Table S10. 5‐byte version 2 Read codes used to identify the presence of left ventricular systolic dysfunction.
Table S11. 5‐byte version 2 Read codes used to identify the presence of left ventricular diastolic dysfunction.
Table S12. 5‐byte version 2 Read codes used to identify the presence of ankle‐brachial index.
Table S13. A comparison of the LEADER trial cardiovascular disease/risk inclusion criteria with the closest matching variables available from routine UK primary care data.
