# Supplementary material for: Real‐world prevalence of the inclusion criteria for the LEADER trial: Data from a national general practice network
Source: Diabetes Obes Metab. 2019 Apr 11;21(7):1661–7. doi: 10.1111/dom.13710 (PMC6619442; doi:10.1111/dom.13710)
Supplement: Supplementary file 1 — Table S1. 5‐byte version 2 Read codes used to identify the presence of myocardial infarction. Table S2. 5‐byte version 2 Read codes used to identify the presence of stroke and transient ischaemic attack. Table S3. 5‐byte version 2 Read codes used to identify the presence of coronary artery disease. Table S4. 5‐byte version 2 Read codes used to identify the presence of unstable angina. Table S5. 5‐byte version 2 Read codes used to identify the presence of chronic heart failure. Table S6. 5‐byte version 2 Read codes used to identify the presence of creatinine. Table S7. 5‐byte version 2 Read codes used to identify the presence of albumin to creatinine ratio. Table S8. 5‐byte version 2 Read codes used to identify the presence of hypertension. Table S9. 5‐byte version 2 Read codes used to identify the presence of left ventricular hypertrophy. Table S10. 5‐byte version 2 Read codes used to identify the presence of left ventricular systolic dysfunction. Table S11. 5‐byte version 2 Read codes used to identify the presence of left ventricular diastolic dysfunction. Table S12. 5‐byte version 2 Read codes used to identify the presence of ankle‐brachial index. Table S13. A comparison of the LEADER trial cardiovascular disease/risk inclusion criteria with the closest matching variables available from routine UK primary care data. [file DOM-21-1661-s001.docx]

**Real-world prevalence of the inclusion criteria for the LEADER trial: data from a national general practice network**

*William Hinton, Michael Feher, Neil Munro, Megan Walker, Simon de Lusignan*

**Supplementary material**

**Table S1. 5-byte version 2 Read codes used to identify the presence of myocardial infarction**

| **Read code** | **Clinical term** |
| --- | --- |
| 323.. | ECG: myocardial infarction |
| 3232 | ECG: old myocardial infarction |
| 3233 | ECG: antero-septal infarct |
| 3234 | ECG: posterior/inferior infarct |
| 3235 | ECG: subendocardial infarct |
| 3236 | ECG: lateral infarction |
| 323Z. | ECG: myocardial infarct NOS |
| G30.. | Acute myocardial infarction |
| G300. | Acute anterolateral infarction |
| G301. | Other specified anterior myocardial infarction |
| G3010 | Acute anteroapical infarction |
| G3011 | Acute anteroseptal infarction |
| G301z | Anterior myocardial infarction NOS |
| G302. | Acute inferolateral infarction |
| G303. | Acute inferoposterior infarction |
| G304. | Posterior myocardial infarction NOS |
| G305. | Lateral myocardial infarction NOS |
| G306. | True posterior myocardial infarction |
| G307. | Acute subendocardial infarction |
| G3070 | Acute non-Q wave infarction |
| G3071 | Acute non-ST segment elevation myocardial infarction |
| G308. | Inferior myocardial infarction NOS |
| G309. | Acute Q-wave infarct |
| G30B. | Acute posterolateral myocardial infarction |
| G30X. | Acute transmural myocardial infarction of unspecified site |
| G30X0 | Acute ST segment elevation myocardial infarction |
| G30y. | Other acute myocardial infarction |
| G30y0 | Acute atrial infarction |
| G30y1 | Acute papillary muscle infarction |
| G30y2 | Acute septal infarction |
| G30yz | Other acute myocardial infarction NOS |
| G30z. | Acute myocardial infarction NOS |
| G310. | Postmyocardial infarction syndrome |
| G32.. | Old myocardial infarction |
| G35.. | Subsequent myocardial infarction |
| G350. | Subsequent myocardial infarction of anterior wall |
| G351. | Subsequent myocardial infarction of inferior wall |
| G353. | Subsequent myocardial infarction of other sites |
| G35X. | Subsequent myocardial infarction of unspecified site |
| G36.. | Certain current complications following acute myocardial infarction |
| G360. | Haemopericardium as current complication following acute myocardial infarction |
| G361. | Atrial septal defect as current complication following acute myocardial infarction |
| G362. | Ventricular septal defect as current complication following acute myocardial infarction |
| G363. | Rupture of cardiac wall without haemopericardium as current complication following acute myocardial infarction |
| G364. | Rupture of chordae tendineae as current complication following acute myocardial infarction |
| G365. | Rupture of papillary muscle as current complication following acute myocardial infarction |
| G366. | Thrombosis of atrium, auricular appendage, and ventricle as current complications following acute myocardial infarction |
| G38.. | Postoperative myocardial infarction |
| G380. | Postoperative transmural myocardial infarction of anterior wall |
| G381. | Postoperative transmural myocardial infarction of inferior wall |
| G382. | Postoperative transmural myocardial infarction of other sites |
| G383. | Postoperative transmural myocardial infarction of unspecified site |
| G384. | Postoperative subendocardial myocardial infarction |
| G38z. | Postoperative myocardial infarction, unspecified |
| 14A3. | H/O: myocardial infarct <60 |
| 14A4. | H/O: myocardial infarct >60 |
| 14AH. | H/O: myocardial infarction in last year |
| 14AT. | History of myocardial infarction |
| 14AW. | H/O acute coronary syndrome |

Term codes were also included for all listed Read codes. H/O, history of; NOS, not otherwise specified.

**Table S2. 5-byte version 2 Read codes used to identify the presence of stroke and transient ischaemic attack**

| **Read code** | **Clinical term** |
| --- | --- |
| G63y0 | Cerebral infarct due to thrombosis of precerebral arteries |
| G63y1 | Cerebral infarction due to embolism of precerebral arteries |
| G64.. | Cerebral arterial occlusion |
| G640. | Cerebral thrombosis |
| G6400 | Cerebral infarction due to thrombosis of cerebral arteries |
| G641. | Cerebral embolism |
| G6410 | Cerebral infarction due to embolism of cerebral arteries |
| G64z. | Cerebral infarction NOS |
| G64z0 | Brainstem infarction |
| G64z1 | Wallenberg syndrome |
| G64z2 | Left sided cerebral infarction |
| G64z3 | Right sided cerebral infarction |
| G64z4 | Infarction of basal ganglia |
| G65.. | Transient cerebral ischaemia |
| G650. | Basilar artery syndrome |
| G651. | Vertebral artery syndrome |
| G6510 | Vertebro-basilar artery syndrome |
| G652. | Subclavian steal syndrome |
| G653. | Carotid artery syndrome hemispheric |
| G654. | Multiple and bilateral precerebral artery syndromes |
| G656. | Vertebrobasilar insufficiency |
| G657. | Carotid territory transient ischaemic attack |
| G65y. | Other transient cerebral ischaemia |
| G65z. | Transient cerebral ischaemia NOS |
| G65z0 | Impending cerebral ischaemia |
| G65z1 | Intermittent cerebral ischaemia |
| G65zz | Transient cerebral ischaemia NOS |
| G66.. | Stroke and cerebrovascular accident unspecified |
| G660. | Middle cerebral artery syndrome |
| G661. | Anterior cerebral artery syndrome |
| G662. | Posterior cerebral artery syndrome |
| G663. | Brain stem stroke syndrome |
| G664. | Cerebellar stroke syndrome |
| G665. | Pure motor lacunar syndrome |
| G666. | Pure sensory lacunar syndrome |
| G667. | Left sided CVA |
| G668. | Right sided CVA |
| G6760 | Cerebral infarction due to cerebral venous thrombosis, nonpyogenic |
| G6W.. | Cerebral infarction due to unspecified occlusion or stenosis of precerebral arteries |
| G6X.. | Cerebral infarction due to unspecified occlusion or stenosis of cerebral arteries |
| Gyu63 | [X]Cerebral infarction due to unspecified occlusion or stenosis of cerebral arteries |
| Gyu64 | [X]Other cerebral infarction |
| Gyu65 | [X]Occlusion and stenosis of other precerebral arteries |
| Gyu66 | [X]Occlusion and stenosis of other cerebral arteries |
| Gyu6G | [X]Cerebral infarction due to unspecified occlusion or stenosis of precerebral arteries |
| Fyu55 | [X]Other transient cerebral ischaemic attacks and related syndromes |
| Fyu56 | [X]Other lacunar syndromes |

Term codes were also included for all listed Read codes. CVA, cerebrovascular accident; NOS, not otherwise specified.

**Table S3. 5-byte version 2 Read codes used to identify the presence of coronary artery disease**

| **Read code** | **Clinical term** |
| --- | --- |
| G3401 | Double coronary vessel disease |
| 792.. | Coronary artery operations |
| 7920 | Saphenous vein graft replacement of coronary artery |
| 79200 | Saphenous vein graft replacement of one coronary artery |
| 79201 | Saphenous vein graft replacement of two coronary arteries |
| 79202 | Saphenous vein graft replacement of three coronary arteries |
| 79203 | Saphenous vein graft replacement of four or more coronary arteries |
| 7920y | Other specified saphenous vein graft replacement of coronary artery |
| 7920z | Saphenous vein graft replacement coronary artery NOS |
| 7921 | Other autograft replacement of coronary artery |
| 79210 | Autograft replacement of one coronary artery NEC |
| 79211 | Autograft replacement of two coronary arteries NEC |
| 79212 | Autograft replacement of three coronary arteries NEC |
| 79213 | Autograft replacement of four of more coronary arteries NEC |
| 7921y | Other specified other autograft replacement of coronary artery |
| 7921z | Other autograft replacement of coronary artery NOS |
| 7922 | Allograft replacement of coronary artery |
| 79220 | Allograft replacement of one coronary artery |
| 79221 | Allograft replacement of two coronary arteries |
| 79222 | Allograft replacement of three coronary arteries |
| 79223 | Allograft replacement of four or more coronary arteries |
| 7922y | Other specified allograft replacement of coronary artery |
| 7922z | Allograft replacement of coronary artery NOS |
| 7923 | Prosthetic replacement of coronary artery |
| 79230 | Prosthetic replacement of one coronary artery |
| 79231 | Prosthetic replacement of two coronary arteries |
| 79232 | Prosthetic replacement of three coronary arteries |
| 79233 | Prosthetic replacement of four or more coronary arteries |
| 7923y | Other specified prosthetic replacement of coronary artery |
| 7923z | Prosthetic replacement of coronary artery NOS |
| 7924 | Revision of bypass for coronary artery |
| 79240 | Revision of bypass for one coronary artery |
| 79241 | Revision of bypass for two coronary arteries |
| 79242 | Revision of bypass for three coronary arteries |
| 79243 | Revision of bypass for four or more coronary arteries |
| 79244 | Revision of connection of thoracic artery to coronary artery |
| 79245 | Revision of implantation of thoracic artery into heart |
| 7924y | Other specified revision of bypass for coronary artery |
| 7924z | Revision of bypass for coronary artery NOS |
| 7925 | Connection of mammary artery to coronary artery |
| 79250 | Double anastomosis of mammary arteries to coronary arteries |
| 79251 | Double implantation of mammary arteries into coronary arteries |
| 79252 | Single anastomosis of mammary artery to left anterior descending coronary artery |
| 79253 | Single anastomosis of mammary artery to coronary artery NEC |
| 79254 | Single implantation of mammary artery into coronary artery |
| 7925y | Other specified connection of mammary artery to coronary artery |
| 7925z | Connection of mammary artery to coronary artery NOS |
| 7926 | Connection of other thoracic artery to coronary artery |
| 79260 | Double anastomosis of thoracic arteries to coronary arteries NEC |
| 79261 | Double implantation of thoracic arteries into coronary arteries NEC |
| 79262 | Single anastomosis of thoracic artery to coronary artery NEC |
| 79263 | Single implantation of thoracic artery into coronary artery NEC |
| 7926y | Other specified connection of other thoracic artery to coronary artery |
| 7926z | Connection of other thoracic artery to coronary artery NOS |
| 7927 | Other open operations on coronary artery |
| 79273 | Transposition of coronary artery NEC |
| 79275 | Open angioplasty of coronary artery |
| 7927y | Other specified other open operation on coronary artery |
| 7927z | Other open operation on coronary artery NOS |
| 7928 | Transluminal balloon angioplasty of coronary artery |
| 79280 | Percutaneous transluminal balloon angioplasty of one coronary artery |
| 79281 | Percutaneous transluminal balloon angioplasty of multiple coronary arteries |
| 79282 | Percutaneous transluminal balloon angioplasty of bypass graft of coronary artery |
| 79283 | Percutaneous transluminal cutting balloon angioplasty of coronary artery |
| 7928y | Other specified transluminal balloon angioplasty of coronary artery |
| 7928z | Transluminal balloon angioplasty of coronary artery NOS |
| 7929 | Other therapeutic transluminal operations on coronary artery |
| 79290 | Percutaneous transluminal laser coronary angioplasty |
| 79293 | Rotary blade coronary angioplasty |
| 79294 | Insertion of coronary artery stent |
| 79295 | Insertion of drug-eluting coronary artery stent |
| 79296 | Percutaneous transluminal atherectomy of coronary artery |
| 7929y | Other specified other therapeutic transluminal operation on coronary artery |
| 7929z | Other therapeutic transluminal operation on coronary artery NOS |
| 792A. | Diagnostic transluminal operations on coronary artery |
| 792A0 | Percutaneous transluminal angioscopy |
| 792Ay | Other specified diagnostic transluminal operation on coronary artery |
| 792Az | Diagnostic transluminal operation on coronary artery NOS |
| 792B. | Repair of coronary artery NEC |
| 792B0 | Endarterectomy of coronary artery NEC |
| 792By | Other specified repair of coronary artery |
| 792Bz | Repair of coronary artery NOS |
| 792C. | Other replacement of coronary artery |
| 792C0 | Replacement of coronary arteries using multiple methods |
| 792Cy | Other specified replacement of coronary artery |
| 792Cz | Replacement of coronary artery NOS |
| 792D. | Other bypass of coronary artery |
| 792Dy | Other specified other bypass of coronary artery |
| 792Dz | Other bypass of coronary artery NOS |
| 792E. | Percutaneous coronary intervention |
| 7.92E+02 | Emergency percutaneous coronary intervention |
| 792y. | Other specified operations on coronary artery |
| 792z. | Coronary artery operations NOS |

Term codes were also included for all listed Read codes. NEC, not elsewhere classified; NOS, not otherwise specified.

**Table S4. 5-byte version 2 Read codes used to identify the presence of unstable angina**

| **Read code** | **Clinical term** |
| --- | --- |
| 662K1 | Angina control – poor |
| 662K3 | Angina control – worsening |
| G3111 | Unstable angina |
| G3112 | Angina at rest |
| G3113 | Refractory angina |
| G3114 | Worsening angina |
| G330. | Angina decubitus |
| G3300 | Nocturnal angina |
| G330z | Angina decubitus NOS |
| G331. | Prinzmetal's angina |

Term codes were also included for all listed Read codes. NOS, not otherwise specified.

**Table S5. 5-byte version 2 Read codes used to identify the presence of chronic heart failure**

| **Read code** | **Clinical term** |
| --- | --- |
| 662f. | New York Heart Association classification – class I |
| 662g. | New York Heart Association classification – class II |
| 662h. | New York Heart Association classification – class III |
| 662i. | New York Heart Association classification – class IV |
| G1yz1 | Rheumatic left ventricular failure |
| G58.. | Heart failure |
| G58.. | Cardiac failure |
| G580. | Congestive heart failure |
| G580. | Congestive cardiac failure |
| G580. | Right heart failure |
| G580. | Right ventricular failure |
| G580. | Biventricular failure |
| G5800 | Acute congestive heart failure |
| G5801 | Chronic congestive heart failure |
| G5802 | Decompensated cardiac failure |
| G5803 | Compensated cardiac failure |
| G5804 | Congestive heart failure due to valvular disease |
| G581. | Left ventricular failure |
| G581. | Asthma – cardiac |
| G581. | Pulmonary oedema – acute |
| G581. | Impaired left ventricular function |
| G5810 | Acute left ventricular failure |
| G582. | Acute heart failure |
| G583. | Heart failure with normal ejection fraction |
| G583. | HFNEF – heart failure with normal ejection fraction |
| G583. | Heart failure with preserved ejection fraction |
| G584. | Right ventricular failure |
| G58z. | Heart failure NOS |
| G58z. | Weak heart |
| G58z. | Cardiac failure NOS |
| 14A6. | H/O: heart failure |
| 14AM. | H/O: heart failure in last year |
| 14S3. | H/O: heart recipient |
| 14T7. | H/O: artificial heart |
| 1O1.. | Heart failure confirmed |
| 662p. | Heart failure 6-month review |
| 662T. | Congestive heart failure monitoring |
| 662W. | Heart failure annual review |
| 679X. | Heart failure education |
| 679W1 | Education about deteriorating heart failure |
| 67D4. | Heart failure information given to patient |
| 7900 | Transplantation of heart and lung |
| 79000 | Allotransplantation of heart and lung |
| 79001 | Revision of transplantation of heart and lung |
| 7900y | Other specified transplantation of heart and lung |
| 7900z | Transplantation of heart and lung NOS |
| 7901 | Other transplantation of heart |
| 79010 | Allotransplantation of heart NEC |
| 79011 | Xenotransplantation of heart |
| 79013 | Piggyback transplantation of heart |
| 79014 | Revision of implantation of prosthetic heart |
| 79015 | Revision of transplantation of heart NEC |
| 7901y | Other specified other transplantation of heart |
| 7901z | Other transplantation of heart NOS |
| 79379 | Implantation of biventricular cardiac pacemaker system |
| 7936J | Implantation of intravenous biventricular cardiac pacemaker system |
| 7933 | Transluminal heart assist operations |
| 79330 | Transluminal insertion of pulsation balloon into aorta |
| 79331 | Transluminal insertion of heart assist system NEC |
| 79332 | Transluminal maintenance of heart assist system |
| 79334 | Implantation of ventricular assist device |
| 79335 | Implantation of right ventricular assist device |
| 79336 | Implantation of left ventricular assist device |
| 79337 | Implantation of biventricular assist device |
| 7933y | Other specified transluminal heart assist operation |
| 7933z | Transluminal heart assist operation NOS |
| 793L. | Open heart assist operations |
| 793L0 | Open implantation of ventricular assist device |
| 793Ly | Other specified open heart assist operations |
| 793Lz | Open heart assist operations NOS |
| 8B29. | Cardiac failure therapy |
| 8CeC. | Preferred place of care for next exacerbation of heart failure |
| 8CL3. | Heart failure care plan discussed with patient |
| 8CMK. | Has heart failure management plan |
| 8CMW8 | Heart failure clinical pathway |
| 8H2S. | Admit heart failure emergency |
| 8HBE. | Heart failure follow-up |
| 8HHb. | Referral to heart failure nurse |
| 8HHz. | Referral to heart failure exercise programme |
| 8Hk0. | Referred to heart failure education group |
| 8HTL. | Referral to heart failure clinic |
| 8HTL0 | Referral to rapid access heart failure clinic |
| 9Or0. | Heart failure review completed |
| 9N0k. | Seen in heart failure clinic |
| 9N2p. | Seen by community heart failure nurse |
| G2101 | Malignant hypertensive heart disease with congestive cardiac failure |
| G2111 | Benign hypertensive heart disease with congestive cardiac failure |
| G21z1 | Hypertensive heart disease NOS with congestive cardiac failure |
| G232. | Hypertensive heart and renal disease with (congestive) heart failure |
| G234. | Hypertensive heart and renal disease with both (congestive) heart failure and renal failure |
| Q48y1 | Congenital cardiac failure |
| Q490. | Neonatal cardiac failure |
| SP084 | Heart transplant failure and rejection |
| SP085 | Heart-lung transplant failure and rejection |
| TB000 | Transplantation of heart as the cause of abnormal reaction of patient, or of later complication, without mention of misadventure at the time of operation |
| ZV421 | [V]Heart transplanted |
| ZV45M | [V]Biventricular pacemaker in situ |

Term codes were also included for all listed Read codes. H/O, history of; NEC, not elsewhere classified; NOS, not otherwise specified.

**Table S6. 5-byte version 2 Read codes used to identify the presence of creatinine**

| **Read code** | **Clinical term** |
| --- | --- |
| 44J3. | Serum creatinine |
| 44JF. | Plasma creatinine level |

Term codes were also included for all listed Read codes.

**Table S7. 5-byte version 2 Read codes used to identify the presence of albumin to creatinine ratio**

| **Read code** | **Clinical term** |
| --- | --- |
| 44J7. | Albumin/creatinine ratio |
| 46TC. | Urine albumin:creatinine ratio |
| 46TD. | Urine microalbumin:creatinine ratio |

Term codes were also included for all listed Read codes.

**Table S8. 5-byte version 2 Read codes used to identify the presence of hypertension**

| **Read code** | **Clinical term** |
| --- | --- |
| 14A2. | H/O: hypertension |
| 662b. | Moderate hypertension control |
| 662c. | Hypertension 6-month review |
| 662d. | Hypertension annual review |
| 6627 | Good hypertension control |
| 6628 | Poor hypertension control |
| 662F. | Hypertension treatment – Started |
| 662G. | Hypertensive treatment – Changed |
| 662O. | On treatment for hypertension |
| 662P. | Hypertension monitoring |
| 662P0 | Hypertension 9-month review |
| 662P1 | Telehealth hypertension monitoring |
| 67H8. | Lifestyle advice regarding hypertension |
| 8B26. | Antihypertensive therapy |
| 8HT5. | Referral to hypertension clinic |
| 9N03. | Seen in hypertension clinic |
| 9OI1. | Attends hypertension monitor |
| G2... | Hypertensive disease |
| G2... | BP – hypertensive disease |
| G20.. | Essential hypertension |
| G20.. | High blood pressure |
| G20.. | Primary hypertension |
| G200. | Malignant essential hypertension |
| G201. | Benign essential hypertension |
| G202. | Systolic hypertension |
| G203. | Diastolic hypertension |
| G20z. | Essential hypertension NOS |
| G20z. | Hypertension NOS |
| G21.. | Hypertensive heart disease |
| G210. | Malignant hypertensive heart disease |
| G2100 | Malignant hypertensive heart disease without congestive cardiac failure |
| G2101 | Malignant hypertensive heart disease with congestive cardiac failure |
| G210z | Malignant hypertensive heart disease NOS |
| G211. | Benign hypertensive heart disease |
| G2110 | Benign hypertensive heart disease without congestive cardiac failure |
| G2111 | Benign hypertensive heart disease with congestive cardiac failure |
| G211z | Benign hypertensive heart disease NOS |
| G21z. | Hypertensive heart disease NOS |
| G21z0 | Hypertensive heart disease NOS without congestive cardiac failure |
| G21z0 | Cardiomegaly – hypertensive |
| G21z1 | Hypertensive heart disease NOS with congestive cardiac failure |
| G21zz | Hypertensive heart disease NOS |
| G22.. | Hypertensive renal disease |
| G22.. | Nephrosclerosis |
| G220. | Malignant hypertensive renal disease |
| G221. | Benign hypertensive renal disease |
| G222. | Hypertensive renal disease with renal failure |
| G22z. | Hypertensive renal disease NOS |
| G22z. | Renal hypertension |
| G23.. | Hypertensive heart and renal disease |
| G230. | Malignant hypertensive heart and renal disease |
| G231. | Benign hypertensive heart and renal disease |
| G232. | Hypertensive heart and renal disease with (congestive) heart failure |
| G233. | Hypertensive heart and renal disease with renal failure |
| G234. | Hypertensive heart and renal disease with both (congestive) heart failure and renal failure |
| G23z. | Hypertensive heart and renal disease NOS |
| G24.. | Secondary hypertension |
| G240. | Secondary malignant hypertension |
| G2400 | Secondary malignant renovascular hypertension |
| G240z | Secondary malignant hypertension NOS |
| G241. | Secondary benign hypertension |
| G2410 | Secondary benign renovascular hypertension |
| G241z | Secondary benign hypertension NOS |
| G244. | Hypertension secondary to endocrine disorders |
| G24z. | Secondary hypertension NOS |
| G24z0 | Secondary renovascular hypertension NOS |
| G24zz | Secondary hypertension NOS |
| G25.. | Stage 1 hypertension (NICE - National Institute for Health and Clinical Excellence 2011) |
| G25.. | Stage 1 hypertension |
| G26.. | Severe hypertension (NICE - National Institute for Health and Clinical Excellence 2011) |
| G26.. | Severe hypertension |
| G27.. | Hypertension resistant to drug therapy |
| G28.. | Stage 2 hypertension (NICE - National Institute for Health and Clinical Excellence 2011) |
| G2y.. | Other specified hypertensive disease |
| G2z.. | Hypertensive disease NOS |
| G672. | Hypertensive encephalopathy |
| G672. | Hypertensive crisis |
| F4213 | Hypertensive retinopathy |
| L1200 | Benign essential hypertension complicating pregnancy, childbirth and the puerperium unspecified |
| L122. | Other pre-existing hypertension in preg/childbirth/puerp |
| L1220 | Other pre-existing hypertension complicating pregnancy, childbirth and the puerperium unspecified |
| L1221 | Other pre-existing hypertension complicating pregnancy, childbirth and the puerperium – delivered |
| L1222 | Other pre-existing hypertension complicating pregnancy, childbirth and the puerperium – delivered with postnatal complication |
| L1223 | Other pre-existing hypertension complicating pregnancy, childbirth and the puerperium – not delivered |
| L1224 | Other pre-existing hypertension complicating pregnancy, childbirth and the puerperium with postnatal complication |
| L122z | Other pre-existing hypertension complicating pregnancy, childbirth and the puerperium NOS |
| L128. | Pre-existing hypertension complicating pregnancy, childbirth and puerperium |
| L1280 | Pre-existing hypertensive heart disease complicating pregnancy, childbirth and the puerperium |
| L1281 | Pre-existing hypertensive heart and renal disease complicating pregnancy, childbirth and the puerperium |
| L1282 | Pre-existing secondary hypertension complicating pregnancy, childbirth and puerperium |
| Gyu2. | [X]Hypertensive diseases |
| Gyu20 | [X]Other secondary hypertension |
| Gyu21 | [X]Hypertension secondary to other renal disorders |

Term codes were also included for all listed Read codes. H/O, history of; NOS, not otherwise specified.

**Table S9. 5-byte version 2 Read codes used to identify the presence of left ventricular hypertrophy**

| **Read code** | **Clinical term** |
| --- | --- |
| G5y34 | Left ventricular hypertrophy |
| 3242. | ECG: shows LVH |
| 324Z. | ECG: LVH NOS |

Term codes were also included for all listed Read codes. ECG, electrocardiogram; LVH. left ventricular hypertrophy; NOS, not otherwise specified.

**Table S10. 5-byte version 2 Read codes used to identify the presence of left ventricular systolic dysfunction**

| **Read code** | **Clinical term** |
| --- | --- |
| 585f. | Echocardiogram shows left ventricular systolic dysfunction |
| G5yy9 | Left ventricular systolic dysfunction |

Term codes were also included for all listed Read codes.

**Table S11. 5-byte version 2 Read codes used to identify the presence of left ventricular diastolic dysfunction**

| **Read code** | **Clinical term** |
| --- | --- |
| 585g. | Echocardiogram shows left ventricular diastolic dysfunction |
| G5yyA | Left ventricular diastolic dysfunction |

Term codes were also included for all listed Read codes.

**Table S12. 5-byte version 2 Read codes used to identify the presence of ankle-brachial index**

| **Read code** | **Clinical term** |
| --- | --- |
| 585a. | ABPI – ankle-brachial pressure index |

Term codes were also included for all listed Read codes.

**Table S13. A comparison of the LEADER trial cardiovascular disease/risk inclusion criteria with the closest matching variables available from routine UK primary care data**

| **LEADER trial inclusion criteria^1^** | **Closest match using routinely collected**  **primary care data** |
| --- | --- |
| **Myocardial infarction** | |
| Prior myocardial infarction event | Coding of a myocardial infarction or other definite indicator of a myocardial infarction |
| **Stroke or TIA** | |
| Prior ischaemic stroke, haemorrhagic stroke, undetermined stroke, stroke disability or TIA | Coding of stroke (ischaemic or haemorrhagic) and TIA |
| **Coronary revascularization** | |
| Percutaneous coronary intervention or coronary artery bypass grafting | Coding for all coronary artery disease procedures (as shown below) |
| **>50% stenosis of coronary, carotid or lower extremity arteries** | |
| >50% stenosis of coronary, carotid, or lower extremity arteries | Coronary artery disease codes documented by previous revascularization (percutaneous transluminal coronary angioplasty ± stent or coronary artery bypass graft) of the coronary arteries  Coding for peripheral artery disease as documented by:   - Limb angioplasty, stenting, or bypass surgery - Limb or foot amputation - Claudication or peripheral gangrene of lower limb(s) |
| **Documented symptomatic coronary heart disease** | |
| Documented positive exercise stress test or any cardiac imaging or unstable angina with ECG changes | Coding for unstable angina that shows poor angina control |
| **Documented asymptomatic cardiac ischaemia** | |
| Documented by positive nuclear imaging test, exercise test or dobutamine stress echocardiogram | Not recorded in primary care data |
| **Chronic heart failure** | |
| New York Heart Association class II – III | Coding for signs/symptoms, procedures, and diagnoses for heart failure |
| **Chronic kidney disease (stage 3 or greater)** | |
| eGFR <60 mL/min/1.73m^2^ per Modification of Diet in Renal Disease formula or <60mL/min/1.73m^2^ per Cockcroft-Gault formula | Coding for creatinine with eGFR test calculated from creatinine reading, with a result <60 mL/min/1.73m^2^. ^2^ |
| **Microalbuminuria or proteinuria** | |
| Microalbuminuria or proteinuria | Coding for albumin creatinine ratio, with a reading ≥3 mg/mmol to denote presence of microalbuminuria |
| **Hypertension and left ventricular hypertrophy** | |
| Hypertension and left ventricular hypertrophy by ECG or imaging | Coding to denote diagnosis of hypertension and left ventricular hypertrophy |
| **Left ventricular systolic or diastolic dysfunction** | |
| Left ventricular systolic or diastolic dysfunction by imaging | Coding to show presence of Left ventricular systolic or diastolic dysfunction |
| **Ankle-brachial index <0.9** | |
| Ankle-brachial index <0.9 | Coding for ankle-brachial index, and those with value <0.9 |

ECG, electrocardiogram; eGFR, estimated glomerular filtration rate; TIA, transient ischaemic attack.

**References**

1. Marso SP, Daniels GH, Brown-Frandsen K, et al. Liraglutide and cardiovascular outcomes in type 2 diabetes. *N Engl J Med* 2016;375:311-322.

2. Cole NI, Liyanage H, Suckling RJ, et al. An ontological approach to identifying cases of chronic kidney disease from routine primary care data: a cross-sectional study. *BMC Nephrol* 2018;19:85.
